# Correction: Association between self-administrated prophylactics and SARS-CoV-2 infection among traditional market vendors from the Central Highlands of Peru: A nested case-control study

**DOI:** 10.1371/journal.pone.0335549

**Published:** 2025-10-27

**Authors:** Daniel A. Andrade, Ana Ho-Palma, Cesar A. Valdivia-Carrera, Astrid Munguia, Christine Leyns, Javier Guitian, Eloy Gonzales-Gustavson

In Fig 3, the group labels “With booster dose” and “Without booster dose” in the bottom of the figure were incorrectly switched. Please see the correct Fig 3 here.

**Fig 3:**
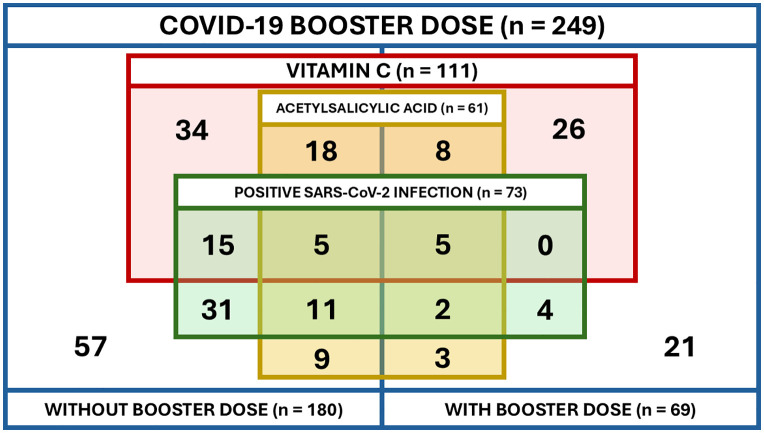
Distribution of participants by variables presenting the main effects of the analysis.
