# Supplementary material for: Association between self-administrated prophylactics and SARS-CoV-2 infection among traditional market vendors from the Central Highlands of Peru: A nested case-control study
Source: PLoS One. 2025 Jul 11;20(7):e0327746. doi: 10.1371/journal.pone.0327746 (PMC12250348; doi:10.1371/journal.pone.0327746)
Supplement: S1 Appendix — (PDF) [file pone.0327746.s001.pdf]

S1 Appendix. Questionnaire administered at the registration of the “Mercado saludable” program.

Supplement to: **“Association between self-administrated prophylactics and SARS-CoV-2 infection among traditional market vendors from the Central Highlands of Peru: A nested case-control study”**

Ficha de Salud

Datos del participante:

1. **Nombres y Apellidos:** ___________________________________
2. **DNI:** _______________
3. **Código del paciente (ejemplo 001):** ______________
4. **Edad:** __________
5. **Número de Celular:** __________________
6. **Género:** Femenino ______ Masculino______ Otro ______

I. Datos clínicos:

Instrucciones: Invitar al participante al tópico para que puedan hacer las siguientes mediciones:

	Primer control	Segundo control	Tercer control	Cuarto control
Fecha (dd/mm/aa)				
Peso (Kg)				
Presión arterial (S/D mmHg)				
IMC				
Porcentaje de grasa (%)				
Perímetro de cintura (cm)				
Temperatura (°C)				
Saturación de oxígeno (%)				
Pulso (Lpm)				
Talla (metros)				
Glucosa (mg/dL) – con el Glucómetro				

II. Análisis Clínicos:

ANÁLISIS DE ORINA

Instrucciones: Indique al participante que vaya al baño y recolecte una muestra de orina fresca (mitad del frasco aproximadamente). Enseguida inserte la tira reactiva de orina en la muestra fresca por segundo, seque el exceso de orina en un papel y espere 30 – 60 segundos para el resultado (60-120 segundos para Leucocitos) y anote el resultado a continuación:

**ANÁLISIS DE ORINA (Hacer un círculo alrededor del resultado correspondiente)**					Fecha (dd/mm/aa)		
**Leucocitos (Leu/µL)**	Negativo	Ca. 25	Ca. 75	Ca. 500			
**Densidad (SG)**	1.000	1.005	1.010	1.015	1.020	1.025	1.030
**pH**	5	6	6.5	7	8	9	

Ficha de Salud

**Glucosa (mg/dL)**	Negativo	Normal	50	150	500	≥ 1000	
**Cetonas**	Negativo	+	++	+++			
**Nitritos**	Negativo	Positivo, cualquier color rosado					
**Proteínas (mg/dL)**	Negativo	Trazas	30	100	500		
**Bilirrubina**	Negativo	+	++	+++			
**Urobilinógeno (mg/dL)**	Normal	2	4	8	12		
**Sangre (Eritrocitos/ µL)**	Negativo	Ca. 10	Ca. 50	Ca. 250	Ca. 5-10	Ca. 50	Ca. 250

Nota: Los valores normales son los de las casillas grises

ANÁLISIS HEMATOLÓGICOS, BIOQUÍMICOS Y COVID-19

Instrucciones: Para los análisis bioquímicos y hematológicos se deberá recolectar la sangre del participante, invítelo a que acuda el día programado para la toma de muestras de sangre. Se necesita un tubo lila (6mL) y un tubo rojo (4mL) por cada participante que deberán enviar al laboratorio de la UNCP para su análisis. Cuando se tenga los resultados, anotarlos en los siguientes cuadros:

ANÁLISIS HEMATOLÓGICOS	Primer control	Segundo control	Tercer control
Fecha (dd/mm/aa)			
Hemoglobina			
Leucocitos			
Linfocitos			
Monocitos			
Plaquetas			
Hematocrito			

ANÁLISIS BIOQUÍMICOS	Primer control	Segundo control	Tercer control
Fecha (dd/mm/aa)			
Glucosa			
Colesterol Total			
Triglicéridos			
Creatinina			
HDL			
Hemoglobina glucosilada (sólo para los que tienen hiperglucemia)			

Instrucciones: Para la prueba de antígeno se deberá sacar una muestra de hisopado nasofaríngeo y analizar la muestra inmediatamente después con el kit de antígeno para SARS-CoV-2. Para la prueba molecular se deberá sacar una muestra de hisopado nasofaríngeo y embalar la muestra teniendo en cuenta todas las medidas de bioseguridad para ser transportadas inmediatamente al laboratorio (estos resultados tardan 5 días en salir).

Ficha de Salud

ANÁLISIS COVID-19	Primer control	Segundo control	Tercer control
Prueba de Antígeno			
Fecha (dd/mm/aa)			
Resultado			
Prueba Molecular			
Fecha (dd/mm/aa)			
Resultado			

III. Historia clínica:

1. **¿Qué familiares tienen alguna de estas enfermedades?**
   Escribir en las casillas el parentesco del familiar que la presenta

Enfermedad	Familiar 1	Familiar 2	Familiar 3	Familiar 4
Obesidad				
Diabetes tipo I				
Diabetes tipo II				
Hipertensión arterial				
Enfermedades cardiovasculares				
Colesterol elevado				
Alergias o asma				
Cáncer				
TBC (tuberculosis)				
Otro: __________________				

2. **¿Usted tiene alguna enfermedad?**

Marca la respuesta indicada por el participante	SI	NO
Diabetes/problema del azúcar		
Del corazón		
De riñones		
Estreñimiento		
artritis/artrosis (problemas articulares)		
Presión alta/hipertensión		
De pulmones		
De hígado		
Enfermedades parasitarias (áscaris, hidatidosis, cisticercosis, criptosporidium, giardia, fasciola). Si la respuesta es afirmativa, indicar el parásito		
Asma		
¿Algún tipo de alergia? Si la respuesta es sí ¿A qué? ______________		
Otro: __________________		

Ficha de Salud

3. ¿Usted ha tenido alguna cirugía en su vida? Si _______ No _______

Si la respuesta es Si, completar el siguiente cuadro:

¿Qué cirugía ha tenido?	SI	NO
Vesícula		
Apéndice		
Cesárea		
Fractura		
Otra: __________________		

4. ¿Usted fuma cigarrillos?

Marca la respuesta indicada por el participante	SI	NO
Fuma		
Fumador a diario		
Si la respuesta es Si preguntar ¿Cuántos cigarrillos aprox. fuma por día?		
Fumador opcional/ social		
Exfumador		

5. ¿Con que frecuencia toma alcohol?

Marcar sólo una respuesta	
No tomo alcohol	
Todos los días de la semana	
Solo los fines de semana	
1-2 veces a la semana	
3-4 veces a la semana	
4-5 veces a la semana	
1 vez al mes	
Otro: _______________	

6. ¿Este año acudió al médico?

Marcar la respuesta	SI	NO
Acudió		
Sí, para revisión general preventiva		
Sí, cuando estaba enfermo		

Si seleccionó "si, cuando estaba enfermo":

¿Cuál era la enfermedad? ________________________________

¿Realiza algún tipo de tratamiento para ello? Como medicamentos, hierbas medicinales, vitaminas u otros ____

Ficha de Salud

7. ¿A qué centro de salud acude usted o un algún miembro de su familia cuando se enferma?

Marcar la respuesta	SI	NO	Para qué enfermedad
Servicios públicos de salud de Huancayo			
Servicios públicos en otro lugar (Especificar lugar)			
Seguridad Social			
Centro/ médico privado			
Medico tradicional			
Farmacia			
Remedios caseros			
Ninguno			

IV. Sección Covid-19:

1. Durante la pandemia, ¿ha consumido alguno de los siguientes alimentos o plantas medicinales para prevenir el COVID 19?

Marcar la respuesta	SI	NO
Eucalipto		
Matico		
Manzanilla		
Molle		
Miel de abeja		
Kion/Jengibre		
Propóleo		
Ajo		
Cebolla		
Wira wira		
Mate de hierbas		
Ninguno		
Otro: _______________		

2. Durante la pandemia, ¿ha consumido alguno de los siguientes medicamentos para prevenir el COVID 19?

Marcar la respuesta	SI	NO
Aspirina		
Ivermectina		
Dióxido de cloro		
Paracetamol		
Ibuprofeno		
hidrocortisona/prednisona/dexametasona/...		
azitromicina/claritromicina		
penicilina/amoxicilina/ceftriaxona		
ciprofloxacina/levofloxacina		

Ficha de Salud

enoxaparina		
N acetilcisteína		
Complejo B		
Zinc		
Vitamina D		
Vitamina C		
Ninguno		
Otro: _______________		

3. **Desde que inició la pandemia, ¿Se ha realizado alguna prueba para COVID 19?**

**Marcar la respuesta**		No	Si
**Si marco Sí ¿Cuántas veces se ha realizado una prueba COVID 19?**			
Indicar nombre de la prueba realizada (antígeno, molecular, serológica o no sabe)	Fecha de la prueba [dd/mm/aa]	Resultado Positivo	Resultado Negativo

4. **¿Usted piensa que ha tenido COVID-19?**
Sólo para personas que marcaron no, en la pregunta 3.

**A. Marcar la respuesta**	No	Si	No sabe
**B. Si marco Sí ¿Cuál es la razón principal por la que piensa que tuvo COVID 19?**			
Tuvo síntomas		No	Si
Un miembro del hogar tuvo COVID 19		No	Si
Contacto con otro familiar o amistad que tuvo COVID 19		No	Si
Otro: ____		No	Si
**C. Si respondió que tuvo síntomas, ¿Qué síntomas de COVID19 tuvo?**			
Temperatura		No	Si
Dolor de garganta		No	Si
Pérdida de gusto u olfato		No	Si
Dificultad para respirar		No	Si
Dolor de cuerpo		No	Si
Diarrea		No	Si
Dolor de cabeza		No	Si
Otros:____		No	Si

Ficha de Salud

5. ¿Qué tipo de tratamiento hizo para curar los síntomas de COVID 19?
Sólo si respondió que tuvo síntomas en la pregunta 4C.

A. Tratamiento con medicamentos:

____

Recetado por un médico: Si_____ No ______

B. Tratamiento natural ¿Qué tipo de medicina natural tomó?:

____

6. ¿Cuáles fueron sus resultados después de aplicar el tratamiento para sus síntomas de COVID 19? *Sólo si respondió que tuvo síntomas en la pregunta 4C.*

**Marcar la respuesta**	Si	No
Se sanó porque estuvo hospitalizado y recibió el tratamiento		
Todavía tiene molestias		
Se sanó porque fue al médico y recibió el tratamiento		

7. ¿Dónde y cómo cree que se contagió?
Sólo si sabe que dio positivo a alguna prueba covid-19 o piensa que tuvo covid-19

**Marcar la respuesta**	Si	No
En el mercado		
En su vivienda		
En la calle		
En el trasporte público		
En una fiesta		
Otro lugar: ____________________________		
No sabe qué lugar		
Por contacto con algún miembro del hogar que tuvo COVID 19		
Por contacto con algún otro familiar o amistad que tuvo COVID 19		
Por contacto con persona extraña		
Otra razón: _______________________________		
No sabe cómo se contagió		

8. Sólo si dio positivo a COVID 19, ¿Ha tenido secuelas después de tener COVID 19?

Si ______ No______

¿Cuáles fueron las secuelas que tuvo?

____

<u>Ficha de Salud</u>

9. Actualmente, ¿Considera usted que ….. ?

Marcar la respuesta	Si	No
Tiene un riesgo **ALTO** de contagiarse de COVID 19		
Tiene un riesgo **MEDIO** de contagiarse de COVID 19		
Tiene un riesgo **BAJO** de contagiarse de COVID 19		
No tiene **NINGÚN RIESGO** de contagiarse de COVID 19		

10. ¿Se ha vacunado? Si ______ No______

¿Qué fecha aproximadamente? ___________________________________

¿Cuál es el nombre de la vacuna? ___________________________________

¿Cuántas dosis ha recibido? ____

11. ¿Algún miembro de su hogar ha dado positivo a COVID 19?

Si ______ No______ Vive solo (a) ______

Continuar con las preguntas sólo si responde que sí en la pregunta 11, caso contrario terminar con el cuestionario.

12. ¿Cuáles son los miembros del hogar que han tenido COVID 19?

**Marcar la respuesta**			**Fecha (mm/aa)**	**Se realizó la prueba COVID 19**		**Indicar con un círculo el estado actual del miembro**		
A.	Esposo (a)			Si	No	Se sanó	Aún tiene molestias	Falleció
B.	Hijo (a) 1			Si	No	Se sanó	Aún tiene molestias	Falleció
C.	Hijo (a) 2			Si	No	Se sanó	Aún tiene molestias	Falleció
D.	Hijo (a) 2			Si	No	Se sanó	Aún tiene molestias	Falleció
E.	Yerno o nuera			Si	No	Se sanó	Aún tiene molestias	Falleció
F.	Padres o suegros			Si	No	Se sanó	Aún tiene molestias	Falleció
G.	Hermano 1			Si	No	Se sanó	Aún tiene molestias	Falleció
H.	Hermano 2			Si	No	Se sanó	Aún tiene molestias	Falleció
I.	Hermano 3			Si	No	Se sanó	Aún tiene molestias	Falleció
J.	Otros parientes			Si	No	Se sanó	Aún tiene molestias	Falleció
K.	Otros no parientes			Si	No	Se sanó	Aún tiene molestias	Falleció
