# Supplementary material for: Association between self-administrated prophylactics and SARS-CoV-2 infection among traditional market vendors from the Central Highlands of Peru: A nested case-control study
Source: PLoS One. 2025 Jul 11;20(7):e0327746. doi: 10.1371/journal.pone.0327746 (PMC12250348; doi:10.1371/journal.pone.0327746)
Supplement: S2 Appendix — (PDF) [file pone.0327746.s002.pdf]

S2 Appendix. Building process of conditional logistic regression adjusted model.

Supplement to: **"Association between self-administrated prophylactics and SARS-CoV-2 infection among traditional market vendors from the Central Highlands of Peru: A nested case-control study"**

Variables	Coefficient	p-value	Percentual variation with previous model	AIC	Likelihood ratio test with previous model (p-value)
Model without interaction effects 1					
Hypertension (Yes)	-1.549	0.020		164.6	
Obesity (Yes)	0.007	0.986			
Self-perceived risk of COVID-19 (Medium)	0.733	0.153			
Self-perceived risk of COVID-19 (Low)	1.016	0.070			
Self-perceived risk of COVID-19 (None)	0.443	0.539			
COVID-19 booster (No)	1.216	0.005			
Acetylsalicylic acid (Yes)	0.849	0.016			
Vitamin C (Yes)	-0.811	0.017			
Model without interaction effects 2					
Hypertension (Yes)	-1.454	0.026	6.1%	168.6	0.014
Obesity (Yes)	0.014	0.970	100%		
Self-perceived risk of COVID-19 (Medium)	0.722	0.148	1.5%		
Self-perceived risk of COVID-19 (Low)	0.986	0.072	3.0%		
Self-perceived risk of COVID-19 (None)	0.541	0.450	22.1%		
COVID-19 booster (No)	1.238	0.003	1.8%		
Acetylsalicylic acid (Yes)	0.673	0.041	20.7%		

The initial model (**Model without Interaction Effects 1**) included all predictors that showed strong statistical associations (p-value < 0.05). However, the predictor with the weakest association—**Vitamin C**—was removed to assess its influence on the model. This exclusion led to

changes greater than 5% in the coefficients of the remaining variables, and the likelihood-ratio test was not statistically significant. Given these variations, the initial model was retained, and the development of a model incorporating interaction effects was pursued.

Variables	Coefficient	p-value	AIC	Likelihood ratio test with previous model (p-value)
Model with interaction effects 1				
Hypertension (Yes)	-1.524	0.024	166.4	
Obesity (Yes)	-0.053	0.894		
Self-perceived risk of COVID-19 (Medium)	0.748	0.161		
Self-perceived risk of COVID-19 (Low)	1.026	0.078		
Self-perceived risk of COVID-19 (None)	0.430	0.577		
COVID-19 booster (No)	1.618	0.016		
Acetylsalicylic acid (Yes)	2.496	0.011		
Vitamin C (Yes)	-1.238	0.196		
COVID-19 booster * Acetylsalicylic acid	-1.927	0.049		
COVID-19 booster * Vitamin C	0.575	0.548		
Acetylsalicylic acid * Vitamin C	-0.172	0.817		
Model with interaction effects 2				
Hypertension (Yes)	-1.526	0.023	164.7	0.817
Obesity (Yes)	-0.039	0.922		
Self-perceived risk of COVID-19 (Medium)	0.767	0.144		
Self-perceived risk of COVID-19 (Low)	1.045	0.069		
Self-perceived risk of COVID-19 (None)	0.473	0.526		
COVID-19 booster (No)	1.618	0.015		
Acetylsalicylic acid (Yes)	2.407	0.008		
Vitamin C (Yes)	-1.317	0.145		
COVID-19 booster * Acetylsalicylic acid	-1.910	0.051		

COVID-19 booster * Vitamin C	0.608	0.523		
**Model with interaction effects 3**				
Hypertension (Yes)	-1.515	0.025	162.8	0.515
Obesity (Yes)	-0.032	0.936		
Self-perceived risk of COVID-19 (Medium)	0.767	0.140		
Self-perceived risk of COVID-19 (Low)	1.042	0.068		
Self-perceived risk of COVID-19 (None)	0.477	0.524		
COVID-19 booster (No)	1.831	0.002		
Acetylsalicylic acid (Yes)	2.238	0.007		
Vitamin C (Yes)	-0.791	0.022		
COVID-19 booster * Acetylsalicylic acid	-1.726	0.056		
**Model without interaction effects 1**				
Hypertension (Yes)	-1.549	0.020	164.6	0.052
Obesity (Yes)	0.007	0.986		
Self-perceived risk of COVID-19 (Medium)	0.733	0.153		
Self-perceived risk of COVID-19 (Low)	1.016	0.070		
Self-perceived risk of COVID-19 (None)	0.443	0.539		
COVID-19 booster (No)	1.216	0.005		
Acetylsalicylic acid (Yes)	0.849	0.016		
Vitamin C (Yes)	-0.811	0.017		

The strategy for building a model with interaction effects involved progressively removing interaction terms that lacked strong statistical association, until only those with strong evidence remained. First, the interaction between **Acetylsalicylic acid** and **Vitamin C** was removed, as it showed the weakest association. A likelihood-ratio test confirmed that this interaction did not contribute significant information to the model. Next, the interaction between the **COVID-19 booster** and **Vitamin C** was removed for the same reason, again supported by a non-significant likelihood-ratio test. Finally, the interaction between the **COVID-19 booster** and **Acetylsalicylic acid** was excluded due to the absence of strong evidence of association. When this reduced model was compared to the initial model (**Model without Interaction Effects 1**), the likelihood-ratio test indicated no significant improvement in fit. Therefore, **Model without Interaction Effects 1** was selected as the final adjusted model.
