# Supplementary material for: Association between self-administrated prophylactics and SARS-CoV-2 infection among traditional market vendors from the Central Highlands of Peru: A nested case-control study
Source: PLoS One. 2025 Jul 11;20(7):e0327746. doi: 10.1371/journal.pone.0327746 (PMC12250348; doi:10.1371/journal.pone.0327746)
Supplement: S1 Table — (PDF) [file pone.0327746.s003.pdf]

S1 Table. SARS-CoV-2 diagnosis by vaccination scheme in selected participants.

Supplement to: **"Association between self-administrated prophylactics and SARS-CoV-2 infection among traditional market vendors from the Central Highlands of Peru: A nested case-control study"**

Vaccination scheme	Total of participants (N = 249)	Cases of positive SARS-CoV-2 diagnosis (n = 73) n (%)
Number of doses		
0 doses	6	1 (16.7%)
1 dose: Oxford/AztraZeneca	4	2 (50.0%)
1 dose: Pfizer-BioNTech	2	1 (50.0%)
1 dose: Sinopharm/BIBP	10	4 (40.0%)
2 doses: Oxford/AztraZeneca	6	2 (33.3%)
2 doses: Pfizer-BioNTech	81	26 (32.1%)
2 doses: Sinopharm/BIBP	71	26 (36.6%)
3 doses: Pfizer-BioNTech	50	6 (12.0%)
3 doses: Sinopharm/BIBP and Oxford/AztraZeneca	2	0 (0.00%)
3 doses: Sinopharm/BIBP and Pfizer-BioNTech	17	5 (29.4%)
