# Supplementary material for: Association between self-administrated prophylactics and SARS-CoV-2 infection among traditional market vendors from the Central Highlands of Peru: A nested case-control study
Source: PLoS One. 2025 Jul 11;20(7):e0327746. doi: 10.1371/journal.pone.0327746 (PMC12250348; doi:10.1371/journal.pone.0327746)
Supplement: S2 Table — (PDF) [file pone.0327746.s004.pdf]

S2 Table. Combination of medications consumed to prevent SARS-CoV-2 infection reported by participants.

Supplement to: **"Association between self-administrated prophylactics and SARS-CoV-2 infection among traditional market vendors from the Central Highlands of Peru: A nested case-control study"**

Combination of medications	Frequency	Percentage (%)
None	32	12.9%
Aspirin, B complex, Vitamin D and Vitamin C	1	0.4%
Aspirin, B complex, Zinc, Vitamin D, Vitamin C and Others	1	0.4%
Aspirin, Chlorine dioxide, Paracetamol, Corticoids, B complex and Others	1	0.4%
Aspirin and Ivermectin	1	0.4%
Aspirin, Ivermectin, Antibiotics, Enoxaparin and Vitamin C	1	0.4%
Aspirin, Ivermectin, Chlorine dioxide, Ibuprofen, N-acetylcysteine, B complex, Vitamin D, Vitamin C and Others	1	0.4%
Aspirin, Ivermectin, Chlorine dioxide, paracetamol, Ibuprofen and Antibiotics	1	0.4%
Aspirin, Ivermectin, Chlorine dioxide, paracetamol, Ibuprofen, Corticoids, Antibiotics, B complex and Vitamin C	1	0.4%
Aspirin, Ivermectin, Chlorine dioxide, paracetamol, Ibuprofen, Corticoids, Antibiotics, B complex, Vitamin D and Vitamin C	1	0.4%
Aspirin, Ivermectin, Chlorine dioxide, paracetamol, Ibuprofen, Corticoids, Antibiotics and Vitamin C	1	0.4%
Aspirin, Ivermectin, Chlorine dioxide, paracetamol, Ibuprofen, Corticoids, B complex, Zinc, Vitamin D and Vitamin C	1	0.4%
Aspirin, Ivermectin, Chlorine dioxide, paracetamol, Ibuprofen and Enoxaparin	1	0.4%
Aspirin, Ivermectin and paracetamol	1	0.4%
Aspirin, Ivermectin, paracetamol, B complex, Vitamin D and Others	1	0.4%

Aspirin, Ivermectin, paracetamol, B complex, Vitamin D, Vitamin C and Others	1	0.4%
Aspirin, Ivermectin, paracetamol, Corticoids, Antibiotics, B complex and Vitamin C	1	0.4%
Aspirin, Ivermectin, paracetamol, Corticoids, Antibiotics and Vitamin C	1	0.4%
Aspirin, Ivermectin, paracetamol and Ibuprofen	2	0.8%
Aspirin, Ivermectin, paracetamol, Ibuprofen and Antibiotics	7	2.8%
Aspirin, Ivermectin, paracetamol, Ibuprofen, Antibiotics, N-acetylcysteine and Others	1	0.4%
Aspirin, Ivermectin, paracetamol, Ibuprofen, Antibiotics and B complex	2	0.8%
Aspirin, Ivermectin, paracetamol, Ibuprofen, Antibiotics, B complex and Vitamin C	1	0.4%
Aspirin, Ivermectin, paracetamol, Ibuprofen, Antibiotics and Others	1	0.4%
Aspirin, Ivermectin, paracetamol, Ibuprofen, Antibiotics and Vitamin C	2	0.8%
Aspirin, Ivermectin, paracetamol, Ibuprofen, Antibiotics, Vitamin C and Others	1	0.4%
Aspirin, Ivermectin, paracetamol, Ibuprofen, B complex, Zinc, Vitamin D and Vitamin C	1	0.4%
Aspirin, Ivermectin, paracetamol, Ibuprofen, Corticoids and Antibiotics	2	0.8%
Aspirin, Ivermectin, paracetamol, Ibuprofen, Corticoids, Antibiotics, N-acetylcysteine B complex and Vitamin C	1	0.4%
Aspirin, Ivermectin, paracetamol, Ibuprofen, Corticoids, Antibiotics, N-acetylcysteine, B complex, Zinc, Vitamin D and Vitamin C	3	1.2%
Aspirin, Ivermectin, paracetamol, Ibuprofen, Corticoids, Antibiotics and B complex	1	0.4%
Aspirin, Ivermectin, paracetamol, Ibuprofen, Corticoids, Antibiotics, B complex and Vitamin C	1	0.4%
Aspirin, Ivermectin, paracetamol, Ibuprofen, Corticoids, Antibiotics, B complex, Zinc and Vitamin D	1	0.4%

Aspirin, Ivermectin, paracetamol, Ibuprofen, Corticoids, Antibiotics, B complex, Zinc, Vitamin D and Vitamin C	4	1.6%
Aspirin, Ivermectin, paracetamol, Ibuprofen, Corticoids, Antibiotics, Enoxaparin, B complex, Vitamin D and Vitamin C	1	0.4%
Aspirin, Ivermectin, paracetamol, Ibuprofen, Corticoids, Antibiotics and Vitamin C	2	0.8%
Aspirin, Ivermectin, paracetamol, Ibuprofen, Corticoids, Antibiotics, Vitamin C and Others	1	0.4%
Aspirin, Ivermectin, paracetamol, Ibuprofen and Vitamin C	2	0.8%
Aspirin, Ivermectin, paracetamol, Ibuprofen and Zinc	1	0.4%
Aspirin, Ivermectin, paracetamol and Vitamin C	1	0.4%
Aspirin and paracetamol	1	0.4%
Aspirin, paracetamol, Ibuprofen, Antibiotics and Vitamin C	1	0.4%
Aspirin, paracetamol, Ibuprofen, B complex and Vitamin C	2	0.8%
Aspirin, paracetamol, Zinc, Vitamin D and Vitamin C	1	0.4%
B complex	1	0.4%
B complex, Zinc, Vitamin D and Vitamin C	2	0.8%
Corticoids, Antibiotics, B complex, Zinc, Vitamin D and Vitamin C	1	0.4%
Chlorine dioxide and paracetamol	1	0.4%
Chlorine dioxide, paracetamol and Ibuprofen	1	0.4%
Chlorine dioxide, paracetamol, Ibuprofen, Antibiotics and Vitamin C	1	0.4%
Chlorine dioxide, paracetamol, Ibuprofen, B complex, Vitamin D and Vitamin C	1	0.4%
Chlorine dioxide, paracetamol, Ibuprofen, Corticoids, Antibiotics and B complex	1	0.4%
Chlorine dioxide, paracetamol, Ibuprofen and Others	1	0.4%
Ibuprofen	1	0.4%
Ibuprofen, B complex, Zinc, Vitamin D and Vitamin C	1	0.4%
Ibuprofen and Corticoids	1	0.4%

Ibuprofen, Corticoids, Antibiotics, B complex, Zinc and Vitamin C	1	0.4%
Ivermectin	8	3.2%
Ivermectin, B complex and Others	1	0.4%
Ivermectin, B complex, Vitamin D and Vitamin C	2	0.8%
Ivermectin, B complex, Zinc, Vitamin D and Vitamin C	2	0.8%
Ivermectin, Corticoids, Antibiotics and Vitamin D	1	0.4%
Ivermectin, Chlorine dioxide and paracetamol	1	0.4%
Ivermectin, Chlorine dioxide, paracetamol, N-acetylcysteine, B complex, Zinc, Vitamin D and Vitamin C	1	0.4%
Ivermectin, Chlorine dioxide, paracetamol, Antibiotics, B complex, Zinc, Vitamin C and Others	1	0.4%
Ivermectin, Chlorine dioxide, paracetamol, Ibuprofen, Antibiotics and B complex	1	0.4%
Ivermectin, Chlorine dioxide, paracetamol, Ibuprofen, B complex, Zinc and Vitamin C	1	0.4%
Ivermectin, Chlorine dioxide, paracetamol, Ibuprofen, Corticoids, Antibiotics, B complex, Zinc and Vitamin C	1	0.4%
Ivermectin, Chlorine dioxide, paracetamol, Ibuprofen, Corticoids, Antibiotics and Others	1	0.4%
Ivermectin, Chlorine dioxide and Vitamin C	1	0.4%
Ivermectin, Ibuprofen and Antibiotics	3	1.2%
Ivermectin, Ibuprofen, Corticoids, Antibiotics and B complex	1	0.4%
Ivermectin and Others	2	0.8%
Ivermectin and paracetamol	4	1.6%
Ivermectin, paracetamol and Antibiotics	5	2.0%
Ivermectin, paracetamol, Antibiotics and Others	1	0.4%
Ivermectin, paracetamol, Antibiotics and Vitamin C	5	2.0%
Ivermectin, paracetamol, B complex, Vitamin D and Vitamin C	1	0.4%
Ivermectin, paracetamol, B complex, Zinc, Vitamin D and Vitamin C	1	0.4%

Ivermectin, paracetamol, Corticoids, Antibiotics, N-acetylcysteine, B complex, Zinc, Vitamin D and Vitamin C	1	0.4%
Ivermectin, paracetamol, Corticoids, Antibiotics, B complex, Vitamin D and Vitamin C	1	0.4%
Ivermectin, paracetamol, Corticoids, Antibiotics, B complex, Zinc, Vitamin D and Vitamin C	1	0.4%
Ivermectin, paracetamol, Corticoids, Antibiotics and Others	1	0.4%
Ivermectin, paracetamol and Ibuprofen	9	3.6%
Ivermectin, paracetamol, Ibuprofen, N-acetylcysteine, B complex, Vitamin D and Vitamin C	1	0.4%
Ivermectin, paracetamol, Ibuprofen, N-acetylcysteine, B complex, Zinc, Vitamin D and Vitamin C	1	0.4%
Ivermectin, paracetamol, Ibuprofen and Antibiotics	5	2.0%
Ivermectin, paracetamol, Ibuprofen, Antibiotics, B complex and Vitamin C	2	0.8%
Ivermectin, paracetamol, Ibuprofen, Antibiotics, B complex, Zinc and Vitamin C	1	0.4%
Ivermectin, paracetamol, Ibuprofen, Antibiotics, B complex, Zinc, Vitamin D and Vitamin C	1	0.4%
Ivermectin, paracetamol, Ibuprofen, Antibiotics and Vitamin C	1	0.4%
Ivermectin, paracetamol, Ibuprofen, Antibiotics and Vitamin D	1	0.4%
Ivermectin, paracetamol, Ibuprofen, Antibiotics, Vitamin D and Vitamin C	1	0.4%
Ivermectin, paracetamol, Ibuprofen, B complex, Zinc, Vitamin D and Vitamin C	1	0.4%
Ivermectin, paracetamol, Ibuprofen and Corticoids	1	0.4%
Ivermectin, paracetamol, Ibuprofen, Corticoids and Antibiotics	3	1.2%
Ivermectin, paracetamol, Ibuprofen, Corticoids, Antibiotics, N-acetylcysteine, B complex, Zinc, Vitamin D and Vitamin C	1	0.4%
Ivermectin, paracetamol, Ibuprofen, Corticoids, Antibiotics, B complex, Vitamin D and Vitamin C	2	0.8%

Ivermectin, paracetamol, Ibuprofen, Corticoids, Antibiotics, B complex, Zinc and Vitamin C	1	0.4%
Ivermectin, paracetamol, Ibuprofen, Corticoids, Antibiotics, B complex, Zinc, Vitamin D and Vitamin C	2	0.8%
Ivermectin, paracetamol, Ibuprofen, Corticoids, Antibiotics and Others	1	0.4%
Ivermectin, paracetamol, Ibuprofen, Corticoids, Antibiotics and Vitamin C	1	0.4%
Ivermectin, paracetamol, Ibuprofen, Corticoids, Antibiotics, Zinc, Vitamin D and Vitamin C	1	0.4%
Ivermectin, paracetamol, Ibuprofen, Corticoids, B complex, Vitamin D and Vitamin C	2	0.8%
Ivermectin, paracetamol, Ibuprofen, Corticoids, B complex, Zinc, Vitamin D and Vitamin C	1	0.4%
Ivermectin, paracetamol, Ibuprofen and Vitamin C	1	0.4%
Ivermectin, paracetamol, Ibuprofen, Vitamin D and Vitamin C	2	0.8%
Ivermectin, paracetamol, Ibuprofen, Zinc and Vitamin C	1	0.4%
Ivermectin, paracetamol and Others	2	0.8%
Ivermectin, paracetamol and Vitamin C	2	0.8%
Ivermectin, paracetamol, Vitamin C and Others	1	0.4%
Ivermectin and Vitamin C	5	2.0%
Ivermectin, Zinc and Vitamin C	1	0.4%
Others	1	0.4%
paracetamol	5	2.0%
Paracetamol and Antibiotics	5	2.0%
Paracetamol, Antibiotics, Vitamin D and Vitamin C	1	0.4%
Paracetamol, B complex and Vitamin C	1	0.4%
Paracetamol, B complex, Zinc and Vitamin C	1	0.4%
Paracetamol, Corticoids and Antibiotics	1	0.4%
Paracetamol, Corticoids, Antibiotics, B complex and Vitamin C	1	0.4%
Paracetamol and Ibuprofen	8	3.2%

Paracetamol, Ibuprofen, Antibiotics and B complex	1	0.4%
Paracetamol, Ibuprofen, Antibiotics, B complex, Zinc, Vitamin D and Vitamin C	1	0.4%
Paracetamol, Ibuprofen, Antibiotics and Vitamin C	1	0.4%
Paracetamol, Ibuprofen, B complex, Vitamin D and Vitamin C	1	0.4%
Paracetamol, Ibuprofen, B complex, Zinc, Vitamin D and Vitamin C	1	0.4%
Paracetamol, Ibuprofen, Corticoids, Antibiotics and B complex	1	0.4%
Paracetamol, Ibuprofen and Vitamin C	2	0.8%
Paracetamol, Ibuprofen, Vitamin D and Vitamin C	1	0.4%
Paracetamol and Vitamin C	1	0.4%
Paracetamol, Vitamin C and Others	1	0.4%
Vitamin C	2	0.8%
Zinc, Vitamin D and Vitamin C	1	0.4%
