# Supplementary material for: Association between self-administrated prophylactics and SARS-CoV-2 infection among traditional market vendors from the Central Highlands of Peru: A nested case-control study
Source: PLoS One. 2025 Jul 11;20(7):e0327746. doi: 10.1371/journal.pone.0327746 (PMC12250348; doi:10.1371/journal.pone.0327746)
Supplement: S3 Table — (PDF) [file pone.0327746.s005.pdf]

S3 Table. Correlation matrix analysis with Cramer's V coefficient for the adjusted conditional logistic regression model building.

Supplement to: **"Association between self-administrated prophylactics and SARS-CoV-2 infection among traditional market vendors from the Central Highlands of Peru: A nested case-control study"**

	COVID-19 booster	Vitamin C consumption	N-acetylcysteine consumption	Acetylsalicylic acid consumption	Hypertension	Obesity	Diabetes
**Vitamin C consumption**	Cramer's V = 0.149						
**N-acetylcysteine consumption**	Cramer's V = 0.042	Cramer's V = 0.200*					
**Acetylsalicylic acid consumption**	Cramer's V = 0.023	Cramer's V = 0.166	Cramer's V = 0.150				
**Hypertension**	Cramer's V = 0.064	Cramer's V = 0.038	Cramer's V = 0.077	Cramer's V = 0.004			
**Obesity**	Cramer's V = 0.056	Cramer's V = 0.084	Cramer's V = 0.008	Cramer's V = 0.092	Cramer's V = 0.140		
**Diabetes**	Cramer's V = 0.045	Cramer's V = 0.010	Cramer's V = 0.019	Cramer's V = 0.051	Cramer's V = 0.032	Cramer's V = 0.153	
**Self-perceived risk of COVID-19**	Cramer's V = 0.098	Cramer's V = 0.112	Cramer's V = 0.110	Cramer's V = 0.156	Cramer's V = 0.177	Cramer's V = 0.096	Cramer's V = 0.103

* Cramer's V coefficient compatible with a weak relationship
