# Supplementary material for: Association between self-administrated prophylactics and SARS-CoV-2 infection among traditional market vendors from the Central Highlands of Peru: A nested case-control study
Source: PLoS One. 2025 Jul 11;20(7):e0327746. doi: 10.1371/journal.pone.0327746 (PMC12250348; doi:10.1371/journal.pone.0327746)
Supplement: Attachment — Submitted filename: Response to Reviewers.docx [file pone.0327746.s007.docx]

Dear Editor,

We sincerely thank the reviewers and the editorial team for their thoughtful comments and suggestions, which have greatly contributed to improving our manuscript (PONE-D-25-09334). In response, we have revised the manuscript accordingly and are pleased to submit the updated version for your consideration.

All comments have been addressed in the accompanying document. Our responses are presented in green, and the corresponding edits to the manuscript are shown in red.

We would also like to offer our sincere apologies for a typographical error identified in Table 1. Specifically, the absolute frequencies for the “Hypertension” variable and the relative frequencies for the “Obesity” variable were incorrectly reported. This mistake was due to a mistyping and did not originate from the dataset itself. We have now corrected these values. Importantly, the rest of the analysis remains unaffected, as the underlying data and statistical procedures were not compromised.

**JOURNAL REQUIREMENTS**

When submitting your revision, we need you to address these additional requirements.

1. Please ensure that your manuscript meets PLOS ONE's style requirements, including those for file naming. The PLOS ONE style templates can be found at

<https://journals.plos.org/plosone/s/file?id=wjVg/PLOSOne_formatting_sample_main_body.pdf>

<https://journals.plos.org/plosone/s/file?id=ba62/PLOSOne_formatting_sample_title_authors_affiliations.pdf>

Thank you for your helpful suggestion. We have adjusted the heading levels to align with the required formatting guidelines and corrected the placement of asterisks in Table 1, ensuring they now appear as superscripts. Additionally, we have reviewed and updated the file names to ensure they comply with the submission requirements.

2. Thank you for stating the following financial disclosure:

“This work was supported by the Medical Research Council and the National Institute for Health and Care Research (NIHR) [MR/V028561/1]. DA and EG received partial support of the Fogarty International Center/NIH [Training grant number D43TW00114]. DA also received partial support of the US Centers for Disease Control and Prevention cooperative agreement [Award number 5U01GH00266].”

Please state what role the funders took in the study. If the funders had no role, please state: "The funders had no role in study design, data collection and analysis, decision to publish, or preparation of the manuscript."

If this statement is not correct you must amend it as needed.

Please include this amended Role of Funder statement in your cover letter; we will change the online submission form on your behalf.

Thank you for bringing to our attention the lack of clarity in the financial disclosure. We have now included the following statement to clarify that the funders had no role in our study:

“This work was supported by the Medical Research Council and the National Institute for Health and Care Research (NIHR) [MR/V028561/1]. DA and EG received partial support of the Fogarty International Center/NIH [Training grant number D43TW00114]. DA also received partial support of the US Centers for Disease Control and Prevention cooperative agreement [Award number 5U01GH00266]. The funders had no role in study design, data collection and analysis, decision to publish, or preparation of the manuscript.”

3. We note that Figure 3 in your submission contain copyrighted images. All PLOS content is published under the Creative Commons Attribution License (CC BY 4.0), which means that the manuscript, images, and Supporting Information files will be freely available online, and any third party is permitted to access, download, copy, distribute, and use these materials in any way, even commercially, with proper attribution. For more information, see our copyright guidelines: <http://journals.plos.org/plosone/s/licenses-and-copyright>.

We require you to either (1) present written permission from the copyright holder to publish these figures specifically under the CC BY 4.0 license, or (2) remove the figures from your submission:

a. You may seek permission from the original copyright holder of Figure 3 to publish the content specifically under the CC BY 4.0 license.

We recommend that you contact the original copyright holder with the Content Permission Form (http://journals.plos.org/plosone/s/file?id=7c09/content-permission-form.pdf) and the following text:

“I request permission for the open-access journal PLOS ONE to publish XXX under the Creative Commons Attribution License (CCAL) CC BY 4.0 (http://creativecommons.org/licenses/by/4.0/). Please be aware that this license allows unrestricted use and distribution, even commercially, by third parties. Please reply and provide explicit written permission to publish XXX under a CC BY license and complete the attached form.”

Please upload the completed Content Permission Form or other proof of granted permissions as an "Other" file with your submission.

In the figure caption of the copyrighted figure, please include the following text: “Reprinted from [ref] under a CC BY license, with permission from [name of publisher], original copyright [original copyright year].”

b. If you are unable to obtain permission from the original copyright holder to publish these figures under the CC BY 4.0 license or if the copyright holder’s requirements are incompatible with the CC BY 4.0 license, please either i) remove the figure or ii) supply a replacement figure that complies with the CC BY 4.0 license. Please check copyright information on all replacement figures and update the figure caption with source information. If applicable, please specify in the figure caption text when a figure is similar but not identical to the original image and is therefore for illustrative purposes only.

Thank you for pointing out that Figure 3 contained copyrighted images. We have removed the figure and updated the numbering of the remaining figures accordingly.

4. We note that Figure 1 in your submission contain [map/satellite] images which may be copyrighted. All PLOS content is published under the Creative Commons Attribution License (CC BY 4.0), which means that the manuscript, images, and Supporting Information files will be freely available online, and any third party is permitted to access, download, copy, distribute, and use these materials in any way, even commercially, with proper attribution. For these reasons, we cannot publish previously copyrighted maps or satellite images created using proprietary data, such as Google software (Google Maps, Street View, and Earth). For more information, see our copyright guidelines: <http://journals.plos.org/plosone/s/licenses-and-copyright>.

We require you to either (1) present written permission from the copyright holder to publish these figures specifically under the CC BY 4.0 license, or (2) remove the figures from your submission:

a. You may seek permission from the original copyright holder of Figure 1 to publish the content specifically under the CC BY 4.0 license.

We recommend that you contact the original copyright holder with the Content Permission Form (<http://journals.plos.org/plosone/s/file?id=7c09/content-permission-form.pdf>) and the following text:

“I request permission for the open-access journal PLOS ONE to publish XXX under the Creative Commons Attribution License (CCAL) CC BY 4.0 (http://creativecommons.org/licenses/by/4.0/). Please be aware that this license allows unrestricted use and distribution, even commercially, by third parties. Please reply and provide explicit written permission to publish XXX under a CC BY license and complete the attached form.”

Please upload the completed Content Permission Form or other proof of granted permissions as an "Other" file with your submission.

In the figure caption of the copyrighted figure, please include the following text: “Reprinted from [ref] under a CC BY license, with permission from [name of publisher], original copyright [original copyright year].”

b. If you are unable to obtain permission from the original copyright holder to publish these figures under the CC BY 4.0 license or if the copyright holder’s requirements are incompatible with the CC BY 4.0 license, please either i) remove the figure or ii) supply a replacement figure that complies with the CC BY 4.0 license. Please check copyright information on all replacement figures and update the figure caption with source information. If applicable, please specify in the figure caption text when a figure is similar but not identical to the original image and is therefore for illustrative purposes only.

The following resources for replacing copyrighted map figures may be helpful:

USGS National Map Viewer (public domain): <http://viewer.nationalmap.gov/viewer/>

The Gateway to Astronaut Photography of Earth (public domain): <http://eol.jsc.nasa.gov/sseop/clickmap/>

Maps at the CIA (public domain): <https://www.cia.gov/library/publications/the-world-factbook/index.html> and <https://www.cia.gov/library/publications/cia-maps-publications/index.html>

NASA Earth Observatory (public domain): <http://earthobservatory.nasa.gov/>

Landsat: <http://landsat.visibleearth.nasa.gov/>

USGS EROS (Earth Resources Observatory and Science (EROS) Center) (public domain): [http://eros.usgs.gov/#](http://eros.usgs.gov/)

Natural Earth (public domain): <http://www.naturalearthdata.com/>

Thank you for pointing out the lack of clarity regarding the source of the basemaps used in the creation of Figure 1. The basemaps were obtained from the *Instituto Nacional de Estadística e Informática* and the *Infraestructura de Datos Espaciales del Perú*, both of which provide public domain data. These can be accessed at the following websites, respectively: <https://ide.inei.gob.pe/> and <https://www.idep.gob.pe/>.

To ensure proper attribution in accordance with the journal’s guidelines, we have added the following statement to the legend of Figure 1:

Line 102: “**Fig 1. Map of the province of Huancayo.** The main map presents the streets (dark grey lines) and points the studied wet markets (red dots) and laboratories (blue dots). The map of the Junin region presents the provinces of Huancayo (green) and Jauja (yellow), while the map of Peru presents the Junin region (dark grey). These maps were created using public domain basemaps provided by the “Instituto Nacional de Estadística e Informática” ([https://ide.inei.gob.pe/](https://ide.inei.gob.pe/)) and the “Infraestructura de Datos Espaciales del Perú” ([https://www.idep.gob.pe/](https://www.idep.gob.pe/)).”

5. We note that there is identifying data in the Supporting Information file <S4_table.csv>. Due to the inclusion of these potentially identifying data, we have removed this file from your file inventory. Prior to sharing human research participant data, authors should consult with an ethics committee to ensure data are shared in accordance with participant consent and all applicable local laws.

Data sharing should never compromise participant privacy. It is therefore not appropriate to publicly share personally identifiable data on human research participants. The following are examples of data that should not be shared:

-Name, initials, physical address

-Ages more specific than whole numbers

-Internet protocol (IP) address

-Specific dates (birth dates, death dates, examination dates, etc.)

-Contact information such as phone number or email address

-Location data

-ID numbers that seem specific (long numbers, include initials, titled “Hospital ID”) rather than random (small numbers in numerical order)

Data that are not directly identifying may also be inappropriate to share, as in combination they can become identifying. For example, data collected from a small group of participants, vulnerable populations, or private groups should not be shared if they involve indirect identifiers (such as sex, ethnicity, location, etc.) that may risk the identification of study participants.

Additional guidance on preparing raw data for publication can be found in our Data Policy (https://journals.plos.org/plosone/s/data-availability#loc-human-research-participant-data-and-other-sensitive-data) and in the following article: http://www.bmj.com/content/340/bmj.c181.long.

Please remove or anonymize all personal information, ensure that the data shared are in accordance with participant consent, and re-upload a fully anonymized data set. Please note that spreadsheet columns with personal information must be removed and not hidden as all hidden columns will appear in the published file.

Thank you for highlighting the presence of potentially identifiable information. To ensure the privacy of study participants, we have removed the columns containing ID numbers (“id” and “clinicalrecord”). In addition, we have included the “self-perceived risk of COVID-19” variable, along with the derived variables used in our analysis, to enhance the transparency and reproducibility of our findings. The revised dataset now includes the following columns:

- Market of procedure – “market”
- Age (years) – “age”
- Age group – “age_group”
- Sex – “sex”
- Body Mass Index – “examination_imc”
- Obesity – “obesity”
- Systolic Blood Pressure – “examination_systolic”
- Diastolic Blood Pressure – “examination_diastolic”
- Hypertension – “hypertension”
- Random Blood Sugar – “examination_glucose”
- Diabetes – “diabetes”
- COVID-19 vaccination scheme – “cr_vaccination”
- COVID-19 booster – “booster”
- No self-medication practices – “selfmed_none”
- Acetylsalicylic acid consumption – “cr_selfmed_aspirin”
- Ivermectin consumption – “cr_selfmed_ivermectin”
- Chlorine dioxide consumption – “cr_selfmed_chlorinediox”
- Paracetamol consumption – “cr_selfmed_paracetamol”
- Ibuprofen consumption – “cr_selfmed_ibuprofen”
- Corticosteroids consumption – “cr_selfmed_corticosteroids”
- Azithromycin/clarithromycin consumption – “cr_selfmed_azitromicin”
- Penicillin/amoxicillin/ceftriaxone consumption – “cr_selfmed_penicilin”
- Ciprofloxacin/levofloxacin consumption – “cr_selfmed_ciprofloxacin”
- Antibiotics consumption – “selfmed_antibiotics”
- Enoxaparin consumption – “cr_selfmed_enoxaparin”
- N-acetylcysteine consumption – “cr_selfmed_nacetylcysteine”
- B complex consumption – “cr_selfmed_bcomplex”
- Zinc consumption – “cr_selfmed_zinc”
- Vitamin D consumption – “cr_selfmed_vitamind”
- Vitamin C consumption – “cr_selfmed_vitaminc”
- Other prophylactics consumption – “cr_selfmed_other”
- Self-perceived risk of COVID-19 – “cr_perceivedrisk”
- SARS-CoV-2 diagnosis – “dx_sarscov2”
- Case-control matching group – “match”

Additional Editor Comments:

To further strengthen this valuable contribution, we recommend addressing several key points:

First, regarding essential statistical validation, as noted by Reviewer 2, additional model diagnostics would enhance the findings. We recommend including appropriate goodness-of-fit measures for the conditional logistic regression and assessing potential collinearity among key predictor variables, particularly given the significant associations reported with prophylactic medications.

Thank you for your valuable comments to improve our analysis. In response, we have included the calculation of the Akaike Information Criterion (AIC) for each adjusted model and complemented our model-building strategy with likelihood ratio tests, as detailed below in “Methods” and “Result” section:

In “Methods” section

Line 297: “Then, remaining variables were entered into a model without interactions following a direct approach. Subsequently, predictors with the weakest evidence of association were iteratively removed, assessing changes in estimated coefficients for remaining variables. This process was complemented by likelihood ratio tests, performed using the “lrtest” function from the “lmtest” package in R [22]. If estimated coefficients varied by 5% or more and the likelihood ratio test was significant (p-value < 0.05), the predictor was deemed contributory to the final model and reintroduced. Otherwise, it was considered non-contributory and discarded. This process continued until only predictors with strong evidence of association and significant likelihood ratio test remained. Subsequently, interaction effects between each drug and COVID-19 booster were introduced into the model. Interaction lacking evidence of association were sequentially removed based on the estimated P values and supported by non-significant likelihood ratio tests. The elimination of interaction effects stopped once only those with strong evidence of association remained in the final adjusted model. Furthermore, the Akaike Information Criterion (AIC) was calculated for each adjusted model assessed using the “AIC" function from the “stats” package in R [23].”

In “Results” section

Line 420: “When evaluating the complete model without interaction effects, it was found that all predictors presented strong evidence of association, and these provided significant information to the model through likelihood ratio test (Appendix S2). Also, comorbidities minimally impacted the estimated coefficients (never exceeding 5%) but were retained as potential confounders. Therefore, it was decided not to remove any variable from the model and to continue evaluating the interaction effects between each medication consumption (vitamin C and acetylsalicylic acid) and COVID-19 booster. As a result, it was found that no interaction effect presented strong evidence of association and did not provide significant information to the model through likelihood ratio test (Appendix S2). Thus, it was decided to maintain the model without interaction effects as the final adjusted model which presented an AIC of 164.6.”

The model-building process—including the results of the likelihood ratio tests and the estimated AIC values—is presented in a new supplementary file titled **Appendix S2**, which is attached in this submission.

Finally, regarding the assessment of potential collinearity, we initially included the estimation of the Phi coefficient between variables considered in the adjusted model. However, since the variable “self-perceived risk of COVID-19” was included in the current analysis and comprises four categories, we have updated our collinearity assessment using Cramer’s V coefficient. This measure follows a similar rationale to the Phi coefficient but is more appropriate for variables with more than two categories, such as “self-perceived risk of COVID-19”. Additionally, we have revised the “Methods” and “Results” sections to enhance the clarity of our explanation regarding the collinearity assessment, as detailed below:

In “Methods” section

Line 292: “Regarding the adjusted model building, potential collinearity among the initial variables considered was evaluated using a correlation matrix based on Cramer's V coefficient, calculated with the “CramerV” function from the “DescTools” package in R [21]. If a group of variables presented at least a weak relationship (Cramer’s V ≥ 0.2), the variable with weaker evidence of association with the SARS-CoV-2 diagnosis was discarded.”

In “Results” section

Line 408: “N-acetylcysteine consumption was also excluded based on the detected collinearity with vitamin C through correlation matrix analysis (S3 Table). The remaining variables did not exhibit potential collinearity, as all Cramér’s V coefficients were below 0.2. Thus, the initial model…”

In order to address this suggestion, the following additional references have been incorporated into the revised version of the manuscript:

21. Signorell A. DescTools: Tools for Descriptive Statistics. R Project; 2023. Available: https://CRAN.R-project.org/package=DescTools

22. Zeileis A, Hothorn T. Diagnostic Checking in Regression Relationships. R News. 2002;2: 7–10.

23. R Core Team. R: A Language and Environment for Statistical Computing. Vienna, Austria: R Foundation for Statistical Computing; 2023. Available: https://www.R-project.org/

Second, with respect to risk perception and indication bias, the perceived COVID-19 risk data collected (S1 Appendix, Q9) could help address potential indication bias related to prophylactic medication use. If feasible, we suggest incorporating the perceived risk variable into the analysis to address this bias, as individuals at higher risk may be more likely to take preventive medications. This incorporation could also help explain the unexpected association with acetylsalicylic acid use and understand how risk perception influences both medication use and preventive behaviors. At a minimum, please describe the distribution of risk perception between cases and controls.

Thank you for suggesting the inclusion of the “self-perceived risk of COVID-19” variable to enhance our statistical analysis. Incorporating this variable did not substantially alter the main results of the final adjusted model; in fact, the evidence of association became even stronger. We have included the variable in our analysis in the “Methods” and “Results” sections as follows:

In “Methods” section

Line 257: “Data on sex, market of origin and self-perceived risk of COVID-19 were directly obtained from the questionnaire (S1 Appendix).”

In “Results” section

Line 386: “**Self-perceived risk of COVID-19**

At the time of registration for the health promotion program, 47.8% of participants (119/249) reported a medium level of self-perceived risk of contracting COVID-19. In comparison, 25.7% (64/249) reported a low level of perceived risk, 18.9% (47/249) reported a high level, and 7.6% (19/249) reported no perceived risk of contracting the disease. During the crude analysis, no association was found between levels of self-perceived risk and the odds of SARS-CoV-2 infection (Table 1).”

Finally, Table 1, Figure 5 and Table S3 were updated with the results obtained including this variable. Also, the results of the “Adjusted analysis” section were updated as follows:

Line 430: “Therefore, not having the COVID-19 booster was associated with 3.38-fold increased odds of SARS-CoV-2 infection (aOR 3.38, 95% CI 1.43 – 7.95). Regarding self-administered prophylactics, acetylsalicylic acid consumption was associated with 2.34-fold increased odds of SARS-CoV-2 infection in participants (aOR 2.34, 95% CI 1.17 – 4.66). Finally, vitamin C consumption maintained a protective effect, being associated with 56% reduced odds of SARS-CoV-2 infection (aOR 0.44, 95% CI 0.23 – 0.87).”

Third, as Reviewer 1 noted, enhancing the context would benefit international readers. We recommend adding a brief section that describes the relevant characteristics of COVID-19 waves in Peru during the study period and explains how this context might influence result interpretation. This addition would significantly improve the paper's utility for international audiences.

We appreciate the observation regarding the absence of contextual information about the COVID-19 pandemic in Peru during the period of our study. We have added two paragraphs that describe the pandemic context during the different phases of data collection, which was comprised by two markedly different scenarios. These additions help to contextualize and interpret the findings related to variables such as self-medication practices, self-perceived risk of COVID-19, and receipt of the COVID-19 booster dose, as outlined below:

Line 461: “The data used in our analysis were collected between August 2021 and March 2022, a period during which Peru experienced distinct phases of the COVID-19 pandemic, marked by fluctuations in transmission rates, vaccination coverage, and public health responses. At the onset of the study, in the latter half of 2021, Peru was emerging from its second wave of COVID-19, which had been associated with the highest mortality rate and was primarily driven by the Lambda and Gamma variants [3]. By this time, daily reported cases had declined to an average of approximately 1,000 per day [4]. Concurrently, vaccine availability was beginning to expand, and several public health restrictions remained in effect [25]. These factors may have contributed to the elevated perception of COVID-19 risk observed among participants (66.7% reported a medium to high level of perceived risk). This context may also help explain the high presence of self-medication practices for COVID-19 prevention found in our study, which exceeded previously reported self-medication prevalence up to 51.3% in Peru [7,9].

In contrast, a markedly different context was observed during the second phase of our study in early 2022. At this point, Peru was entering its third wave of COVID-19, characterized by the highest peak of daily reported cases (nearly 60,000), although accompanied by considerably lower mortality rates. This wave was predominantly driven by the Delta and Omicron variants[3]. Daily cases numbers rose sharply from the previously reported average of 1,000 [4]. Additionally, COVID-19 booster doses had become widely available for adults across all age groups, and many restrictions had been relaxed, allowing for a gradual return to pre-pandemic routines [26]. Despite the availability of booster doses, fewer than one-third of participants were protected with the COVID-19 booster dose at the time of their diagnosis during the second phase of our study. This low coverage may be partially explained by reduced vaccine acceptance in several regions of the Central Highlands in Peru [27]. Bendezu-Quispe *et al.* [28] suggest that lower educational attainment may contribute to this pattern, as their study found that not having a postgraduate degree was associated with lower uptake of the third dose. This is particularly relevant given that education indicators in the Highlands regions tend to be lower than those in the coastal areas [29]. Furthermore, the phenomenon known as “pandemic fatigue” -characterized by diminished motivation to adhere to health measures over time- may have contributed to reduced adherence to full vaccination schedules [30,31]. Although the efficacy of the third dose has been demonstrated in various studies [32–35], including our own findings, addressing the underlying causes of reduced vaccination intent remains essential to improving the effectiveness of future immunization campaigns.”

Finally, given that these two markedly different scenarios may have contributed to an overestimation of certain behaviors associated with SARS-CoV-2 diagnosis during the second phase of our study, we have included the following limitation in the “Discussion” section:

Line 551: “Second, our data collection occurred during two markedly different scenarios of the COVID-19 pandemic, as previously described. The information on perceived risk of COVID-19 and self-medication practices was gathered during a period characterized by a high uncertainty regarding vaccine availability and following the most lethal wave of infections and responses were not reconfirmed at later stages of the program. By contrast, participants were diagnosed with SARS-CoV-2 during the third wave, a time when vaccines were widely available and mortality rates had considerably declined. Consequently, it is possible that participants perceptions and behaviours had changed by the time of their diagnosis. This temporal discrepancy may have led to an overestimation of perceived risk and self-medication practices, which should be considered when interpreting the findings of this study.”

In order to address this suggestion, the following additional references have been incorporated into the revised version of the manuscript:

25. Arrasco J, Medina J, Ordoñez L, Vargas Linares E, Ramos W. Características de la mortalidad por COVID-19 durante y después de la pandemia en el Perú. An Fac med. 2024;85: 259–267. doi:10.15381/anales.v85i3.28037

26. Caycho-Rodríguez T, Oré-Kovacs N, Carbajal-León C, Llerena-Espezúa X, Yupanqui-Lorenzo DE, Torales J. Reasons for the use of masks in open areas after the Peruvian government announced that it will no longer be mandatory in the context of COVID-19 in 2022. Medicina Clínica y Social. 2023;7: 61–69. doi:10.52379/mcs.v7i2.289

27. Vizcardo D, Salvador LF, Nole-Vara A, Dávila KP, Alvarez-Risco A, Yáñez JA, et al. Sociodemographic Predictors Associated with the Willingness to Get Vaccinated against COVID-19 in Peru: A Cross-Sectional Survey. Vaccines. 2022;10: 48. doi:10.3390/vaccines10010048

28. Bendezu-Quispe G, Caira-Chuquineyra B, Fernandez-Guzman D, Urrunaga-Pastor D, Herrera-Añazco P, Benites-Zapata VA. Factors Associated with Not Receiving a Booster Dose of COVID-19 Vaccine in Peru. Vaccines (Basel). 2022;10: 1183. doi:10.3390/vaccines10081183

29. Instituto Nacional de Estadística e Informática (INEI). Perú: Indicadores de Educación por Departamento 2009–2019. Lima: Dirección Técnica de Demografía e Indicadores Sociales; Report No.: 319. Available: https://www.inei.gob.pe/media/MenuRecursivo/publicaciones_digitales/Est/Lib1751/libro.pdf

30. Haktanir A, Can N, Seki T, Kurnaz MF, Dilmaç B. Do we experience pandemic fatigue? current state, predictors, and prevention. Curr Psychol. 2022;41: 7314–7325. doi:10.1007/s12144-021-02397-w

31. Meacci L, Primicerio M. Pandemic fatigue impact on COVID-19 spread: A mathematical modelling answer to the Italian scenario. Results in Physics. 2021;31: 104895. doi:10.1016/j.rinp.2021.104895

32. Moreira ED, Kitchin N, Xu X, Dychter SS, Lockhart S, Gurtman A, et al. Safety and Efficacy of a Third Dose of BNT162b2 Covid-19 Vaccine. N Engl J Med. 2022;386: 1910–1921. doi:10.1056/NEJMoa2200674

33. Patalon T, Saciuk Y, Peretz A, Perez G, Lurie Y, Maor Y, et al. Waning effectiveness of the third dose of the BNT162b2 mRNA COVID-19 vaccine. Nat Commun. 2022;13: 3203. doi:10.1038/s41467-022-30884-6

34. Akaishi T, Kushimoto S, Katori Y, Sugawara N, Egusa H, Igarashi K, et al. Effectiveness of third vaccine dose for coronavirus disease 2019 during the Omicron variant pandemic: a prospective observational study in Japan. Sci Rep. 2022;12: 13589. doi:10.1038/s41598-022-17990-7

35. Tan CY, Chiew CJ, Pang D, Lee VJ, Ong B, Lye DC, et al. Protective immunity of SARS-CoV-2 infection and vaccines against medically attended symptomatic omicron BA.4, BA.5, and XBB reinfections in Singapore: a national cohort study. Lancet Infect Dis. 2023;23: 799–805. doi:10.1016/S1473-3099(23)00060-9

Next, in the limitations discussion, your current section appropriately addresses the binary nature of prophylaxis data. We encourage you to maintain this discussion but consider briefly explaining how this limitation might affect the interpretation of the reported associations, along with suggesting key considerations for future research.

We have revised the explanation of this limitation to better clarify its impact on the interpretation of the reported associations and to suggest key considerations for future research, as follows:

Line 573: “Fifth, the self-administered prophylaxis questions assessed only the occurrence of consumption, without capturing details on dosage and frequency. This limitation may introduce information bias, as the dosage and frequency of medication use likely varied widely among participants. Consequently, it is not possible to establish a robust causal relationship between reported consumption and SARS-CoV-2 diagnosis. This should be considered when interpreting the results and highlights the need for more detailed assessments in future research.”

Lastly, we believe that clarifying the contemporary relevance of the findings could enhance the impact of your study. Strengthening the introduction with a brief overview of current public health implications and relevance to future pandemic preparedness would be beneficial.

Thank you for pointing out the lack of clarity regarding the relevance of our study in the current context. In response, we have added a statement highlighting its contemporary relevance in both the “Abstract” and “Introduction” sections, as follows:

In “Abstract” section

Line 31: “Although COVID-19 is no longer a public health emergency of international concern, understanding behaviours such as self-medication remains relevant for informing future outbreak responses and improving public health preparedness. Despite its widespread use during the pandemic, research on medications preventing SARS-CoV-2 infection in healthy individuals is scarce.”

In “Introduction” section

Line 78: “Understanding the association between self-medication and SARS-CoV-2 infection remains important, even though COVID-19 is no longer considered a public health emergency of international concern. Self-medication practices can still affect timely diagnosis, disease progression, and healthcare-seeking behaviour, which are critical for managing ongoing cases and preparing for future respiratory outbreaks [8]. Investigating this relationship provides valuable insights for public health strategies aimed at promoting rational medication use and improving early detection and management of infectious diseases.”

Please prioritize the statistical enhancements suggested by Reviewer 2, while also considering the integration of perceived risk data based on availability and feasibility. Additionally, the temporal context addition need not be extensive, but it should provide sufficient information for proper interpretation.

We are sincerely grateful for the valuable comments which have contributed to improving the quality of our findings. As suggested, we have prioritized enhancing the statistical analysis by incorporating likelihood ratio tests into our model-building strategy, reporting the corresponding AIC estimates in Appendix S2. Additionally, we have updated the results to include data on self-perceived risk of COVID-19 and updating Cramer’s V estimation to assess potential collinearity between variables included in the adjusted model. Finally, we have added relevant contextual information about the COVID-19 pandemic in Peru to support a more accurate interpretation of our findings by an international audience. However, we remain open to reducing or refining this content if necessary to further improve the clarity and focus of the manuscript.

**REVIEWERS’ COMMENTS**

REVIEWER #1

We would like to commend the authors for their rigorous methodological approach to this study. While the study may have been significant during the COVID-19 pandemic, it is not entirely clear why its findings remain relevant in the present context. The introduction should explicitly justify why these data are still important today and how they contribute to current public health discussions.

We appreciate the comment regarding our methodological approach. In response to the suggestion about clarifying the current relevance of our study, we have incorporated the following paragraph in both the “Abstract” and “Introduction” sections:

In “Abstract” section

Line 31: “Although COVID-19 is no longer a public health emergency of international concern, understanding behaviours such as self-medication remains relevant for informing future outbreak responses and improving public health preparedness. Despite its widespread use during the pandemic, research on medications preventing SARS-CoV-2 infection in healthy individuals is scarce.”

In “Introduction” section

Line 78: “Understanding the association between self-medication and SARS-CoV-2 infection remains important, even though COVID-19 is no longer considered a public health emergency of international concern. Self-medication practices can still affect timely diagnosis, disease progression, and healthcare-seeking behaviour, which are critical for managing ongoing cases and preparing for future respiratory outbreaks [8]. Investigating this relationship provides valuable insights for public health strategies aimed at promoting rational medication use and improving early detection and management of infectious diseases.”

Additionally, the study collected data between 2021 and 2022, yet a substantial portion of the survey responses reflect cross-sectional data from 2021. This is particularly important in the Peruvian context, as the characteristics of each COVID-19 wave differed significantly, especially in terms of population practices and behavioral trends. Although the authors acknowledge this as a limitation, further clarification on how this temporal variation may have influenced the results would be beneficial.

Thank you for highlighting the lack of clarification regarding how this limitation may have influenced our results. Given its importance, we have addressed this issue more prominently by presenting it as the second point in our limitations paragraph, incorporating the following ideas:

Line 551: “Second, our data collection occurred during two markedly different scenarios of the COVID-19 pandemic, as previously described. The information on perceived risk of COVID-19 and self-medication practices was gathered during a period characterized by a high uncertainty regarding vaccine availability and following the most lethal wave of infections and responses were not reconfirmed at later stages of the program. By contrast, participants were diagnosed with SARS-CoV-2 during the third wave, a time when vaccines were widely available and mortality rates had considerably declined. Consequently, it is possible that participants perceptions and behaviours had changed by the time of their diagnosis. This temporal discrepancy may have led to an overestimation of perceived risk and self-medication practices, which should be considered when interpreting the findings of this study.”

Furthermore, the study’s findings may not be fully generalizable, not only due to the specific population studied but also because different COVID-19 waves may have exhibited distinct epidemiological and behavioral patterns.

We have revised the wording of our first limitation concerning the generalizability of our findings, incorporating the specific COVID-19 context in which our study was conducted, as follows:

Line 542: “Our study has limitations. First, the results found in our study may not be fully generalizable, as participants were exclusively traditional market vendors. However, traditional markets could serve as potential SARS-CoV-2 sentinel surveillance sites, given its constant interaction with the general population. Additionally, the behavioral patterns observed were influenced by the specific context of the COVID-19 pandemic during the time of data collection. These behaviours may have changed in the current context, in which COVID-19 is no longer considered an international public health emergency. Nonetheless, understanding these patterns remains relevant for guiding preparedness strategies and improving risk communication in future respiratory outbreaks.”

Another key limitation is that the questionnaire only assessed whether participants self-administered prophylactic treatments, without considering dosage or duration of use. This is a crucial factor, as some individuals may have taken prophylactics sporadically (once), while others may have engaged in prolonged or repeated use, leading to potentially different health implications. While it is understandable that the study focuses on self-administration, it is possible that these subgroups differed in ways that could affect the observed associations. This limitation should be further elaborated in the discussion, along with an explanation of how it could impact the study's results.

Thanks for pointing out this lack of information on how this limitation could have affected the observed association. We have clarified its impact on the interpretation of the reported associations and to suggest key considerations for future research, as follows:

Line 573: “Fifth, the self-administered prophylaxis questions assessed only the occurrence of consumption, without capturing details on dosage and frequency. This limitation may introduce information bias, as the dosage and frequency of medication use likely varied widely among participants. Consequently, it is not possible to establish a robust causal relationship between reported consumption and SARS-CoV-2 diagnosis. This should be considered when interpreting the results and highlights the need for more detailed assessments in future research.”

Lastly, we strongly recommend including a dedicated section that contextualizes the COVID-19 situation in Peru at the time of data collection. This would be particularly beneficial for international readers unfamiliar with the Peruvian pandemic timeline, as references to the "third wave" might otherwise be misinterpreted based on the experience of other countries, where variant predominance, timeframes, and vaccination coverage varied. Providing this background would enhance the clarity and applicability of the findings.

We are grateful for the observation highlighting the lack of contextual information about the COVID-19 pandemic in Peru during the study period. In response, we have added two paragraphs describing the pandemic context during the distinct phases of data collection, which occurred under two markedly different scenarios. These additions provide important context for interpreting key findings, particularly those related to self-medication practices, self-perceived risk of COVID-19, and receipt of the COVID-19 booster dose, as detailed below:

Line 461: “The data used in our analysis were collected between August 2021 and March 2022, a period during which Peru experienced distinct phases of the COVID-19 pandemic, marked by fluctuations in transmission rates, vaccination coverage, and public health responses. At the onset of the study, in the latter half of 2021, Peru was emerging from its second wave of COVID-19, which had been associated with the highest mortality rate and was primarily driven by the Lambda and Gamma variants [3]. By this time, daily reported cases had declined to an average of approximately 1,000 per day [4]. Concurrently, vaccine availability was beginning to expand, and several public health restrictions remained in effect [25]. These factors may have contributed to the elevated perception of COVID-19 risk observed among participants (66.7% reported a medium to high level of perceived risk). This context may also help explain the high presence of self-medication practices for COVID-19 prevention found in our study, which exceeded previously reported self-medication prevalence up to 51.3% in Peru [7,9].

In contrast, a markedly different context was observed during the second phase of our study in early 2022. At this point, Peru was entering its third wave of COVID-19, characterized by the highest peak of daily reported cases (nearly 60,000), although accompanied by considerably lower mortality rates. This wave was predominantly driven by the Delta and Omicron variants[3]. Daily cases numbers rose sharply from the previously reported average of 1,000 [4]. Additionally, COVID-19 booster doses had become widely available for adults across all age groups, and many restrictions had been relaxed, allowing for a gradual return to pre-pandemic routines [26]. Despite the availability of booster doses, fewer than one-third of participants were protected with the COVID-19 booster dose at the time of their diagnosis during the second phase of our study. This low coverage may be partially explained by reduced vaccine acceptance in several regions of the Central Highlands in Peru [27]. Bendezu-Quispe *et al.* [28] suggest that lower educational attainment may contribute to this pattern, as their study found that not having a postgraduate degree was associated with lower uptake of the third dose. This is particularly relevant given that education indicators in the Highlands regions tend to be lower than those in the coastal areas [29]. Furthermore, the phenomenon known as “pandemic fatigue” -characterized by diminished motivation to adhere to health measures over time- may have contributed to reduced adherence to full vaccination schedules [30,31]. Although the efficacy of the third dose has been demonstrated in various studies [32–35], including our own findings, addressing the underlying causes of reduced vaccination intent remains essential to improving the effectiveness of future immunization campaigns.”

In order to address this suggestion, the following additional references have been incorporated into the revised version of the manuscript:

25. Arrasco J, Medina J, Ordoñez L, Vargas Linares E, Ramos W. Características de la mortalidad por COVID-19 durante y después de la pandemia en el Perú. An Fac med. 2024;85: 259–267. doi:10.15381/anales.v85i3.28037

26. Caycho-Rodríguez T, Oré-Kovacs N, Carbajal-León C, Llerena-Espezúa X, Yupanqui-Lorenzo DE, Torales J. Reasons for the use of masks in open areas after the Peruvian government announced that it will no longer be mandatory in the context of COVID-19 in 2022. Medicina Clínica y Social. 2023;7: 61–69. doi:10.52379/mcs.v7i2.289

27. Vizcardo D, Salvador LF, Nole-Vara A, Dávila KP, Alvarez-Risco A, Yáñez JA, et al. Sociodemographic Predictors Associated with the Willingness to Get Vaccinated against COVID-19 in Peru: A Cross-Sectional Survey. Vaccines. 2022;10: 48. doi:10.3390/vaccines10010048

28. Bendezu-Quispe G, Caira-Chuquineyra B, Fernandez-Guzman D, Urrunaga-Pastor D, Herrera-Añazco P, Benites-Zapata VA. Factors Associated with Not Receiving a Booster Dose of COVID-19 Vaccine in Peru. Vaccines (Basel). 2022;10: 1183. doi:10.3390/vaccines10081183

29. Instituto Nacional de Estadística e Informática (INEI). Perú: Indicadores de Educación por Departamento 2009–2019. Lima: Dirección Técnica de Demografía e Indicadores Sociales; Report No.: 319. Available: https://www.inei.gob.pe/media/MenuRecursivo/publicaciones_digitales/Est/Lib1751/libro.pdf

30. Haktanir A, Can N, Seki T, Kurnaz MF, Dilmaç B. Do we experience pandemic fatigue? current state, predictors, and prevention. Curr Psychol. 2022;41: 7314–7325. doi:10.1007/s12144-021-02397-w

31. Meacci L, Primicerio M. Pandemic fatigue impact on COVID-19 spread: A mathematical modelling answer to the Italian scenario. Results in Physics. 2021;31: 104895. doi:10.1016/j.rinp.2021.104895

32. Moreira ED, Kitchin N, Xu X, Dychter SS, Lockhart S, Gurtman A, et al. Safety and Efficacy of a Third Dose of BNT162b2 Covid-19 Vaccine. N Engl J Med. 2022;386: 1910–1921. doi:10.1056/NEJMoa2200674

33. Patalon T, Saciuk Y, Peretz A, Perez G, Lurie Y, Maor Y, et al. Waning effectiveness of the third dose of the BNT162b2 mRNA COVID-19 vaccine. Nat Commun. 2022;13: 3203. doi:10.1038/s41467-022-30884-6

34. Akaishi T, Kushimoto S, Katori Y, Sugawara N, Egusa H, Igarashi K, et al. Effectiveness of third vaccine dose for coronavirus disease 2019 during the Omicron variant pandemic: a prospective observational study in Japan. Sci Rep. 2022;12: 13589. doi:10.1038/s41598-022-17990-7

35. Tan CY, Chiew CJ, Pang D, Lee VJ, Ong B, Lye DC, et al. Protective immunity of SARS-CoV-2 infection and vaccines against medically attended symptomatic omicron BA.4, BA.5, and XBB reinfections in Singapore: a national cohort study. Lancet Infect Dis. 2023;23: 799–805. doi:10.1016/S1473-3099(23)00060-9

Finally, the study states that "the employed rapid antigen test has a sensitivity of 96.5% and a specificity of 99.7%." This assertion should be properly cited, as existing literature reports varying values for the sensitivity and specificity of rapid antigen tests. For example, a review published in PLOS presents different estimates. Proper citation of the source for these values is necessary to ensure scientific accuracy and allow readers to critically assess the diagnostic validity of the test used in the study.

We are grateful for the reference to the systematic review provided. Initially, we considered the sensitivity and specificity reported by the test manufacturer. However, we have now updated these parameters based on the findings of the systematic review and have clarified the potential impact of these parameters on the interpretation of our results, as detailed below:

Line 561: “Third, SARS-CoV-2 diagnosis was performed using two different methods due to logistical constraints. According to a systematic review on rapid antigen tests for SARS-CoV-2 [48], the test used in our study reported a sensitivity and specificity of 92.5% and 97.8% when compared with PCR assay, respectively [49]. This difference in diagnostic accuracy may have led to misclassification, particularly underestimating infections during the pre-third wave phase due to the lower sensitivity of the antigen test compared to RT-qPCR. As a result, some participants classified as SARS-CoV-2 negative before the third wave could have been false negatives, which might have affected the estimation of associations between self-administered prophylaxis and subsequent infection. This potential bias should be considered when interpreting the study findings.”

REVIEWER #2

El trabajo aplica correctamente la metodología, sin embargo, pudiera precisar cuál fue la bondad de ajuste para no sobreestimar los resultados como como el test de Hosmer-Lemeshow o AIC/BIC. No se reporta si hubo problemas con la colinealidad de las variables finales.

In order to ensure that our response to this suggestion is clear and aligned with the language in which it was provided, we are presenting our reply first in Spanish, followed by the English version, to maintain full transparency.

**SPANISH ANSWER**

Agradecemos sinceramente sus valiosos comentarios orientados a mejorar nuestro análisis. En respuesta, hemos incorporado el cálculo del Criterio de Información de Akaike (AIC) para cada modelo ajustado y hemos mejorado nuestra estrategia de construcción del modelo mediante la inclusión de pruebas de razón de verosimilitud. Estas actualizaciones se detallan en las secciones de “Métodos” y “Resultados”, respectivamente, como se indica a continuación:

En la sección “Métodos”

Línea 297: “Then, remaining variables were entered into a model without interactions following a direct approach. Subsequently, predictors with the weakest evidence of association were iteratively removed, assessing changes in estimated coefficients for remaining variables. This process was complemented by likelihood ratio tests, performed using the “lrtest” function from the “lmtest” package in R [22]. If estimated coefficients varied by 5% or more and the likelihood ratio test was significant (p-value < 0.05), the predictor was deemed contributory to the final model and reintroduced. Otherwise, it was considered non-contributory and discarded. This process continued until only predictors with strong evidence of association and significant likelihood ratio test remained. Subsequently, interaction effects between each drug and COVID-19 booster were introduced into the model. Interaction lacking evidence of association were sequentially removed based on the estimated P values and supported by non-significant likelihood ratio tests. The elimination of interaction effects stopped once only those with strong evidence of association remained in the final adjusted model. Furthermore, the Akaike Information Criterion (AIC) was calculated for each adjusted model assessed using the “AIC" function from the “stats” package in R [23].”

En la sección “Resultados”

Línea 420: “When evaluating the complete model without interaction effects, it was found that all predictors presented strong evidence of association, and these provided significant information to the model through likelihood ratio test (Appendix S2). Also, comorbidities minimally impacted the estimated coefficients (never exceeding 5%) but were retained as potential confounders. Therefore, it was decided not to remove any variable from the model and to continue evaluating the interaction effects between each medication consumption (vitamin C and acetylsalicylic acid) and COVID-19 booster. As a result, it was found that no interaction effect presented strong evidence of association and did not provide significant information to the model through likelihood ratio test (Appendix S2). Thus, it was decided to maintain the model without interaction effects as the final adjusted model which presented an AIC of 164.6.”

El proceso de construcción del modelo—incluyendo los resultados de las pruebas de razón de verosimilitud y los valores estimados del AIC—se presenta ahora en un nuevo archivo suplementario titulado Apéndice S2, el cual ha sido incluido en esta versión del manuscrito.

Además, con respecto a la evaluación de la posible colinealidad, inicialmente estimamos el coeficiente Phi entre las variables consideradas en el modelo ajustado. Sin embargo, dado que en el análisis actual se ha incluido la variable “percepción del riesgo de contraer COVID-19” (que consta de cuatro categorías), hemos actualizado nuestra evaluación de colinealidad utilizando el coeficiente V de Cramer. Esta medida sigue un razonamiento similar al coeficiente Phi, pero es más adecuada para variables con más de dos categorías. Para mejorar la transparencia, también hemos revisado las secciones de “Métodos” y “Resultados” con el fin de clarificar la explicación de la evaluación de colinealidad, como se detalla a continuación:

En la sección “Métodos”

Línea 292: “Regarding the adjusted model building, potential collinearity among the initial variables considered was evaluated using a correlation matrix based on Cramer's V coefficient, calculated with the “CramerV” function from the “DescTools” package in R [21]. If a group of variables presented at least a weak relationship (Cramer’s V ≥ 0.2), the variable with weaker evidence of association with the SARS-CoV-2 diagnosis was discarded.”

En la sección “Resultados”

Línea 408: “N-acetylcysteine consumption was also excluded based on the detected collinearity with vitamin C through correlation matrix analysis (S3 Table). The remaining variables did not exhibit potential collinearity, as all Cramér’s V coefficients were below 0.2. Thus, the initial model…”

Para atender esta sugerencia, se han incorporado las siguientes referencias adicionales en la versión revisada del manuscrito:

21. Signorell A. DescTools: Tools for Descriptive Statistics. R Project; 2023. Available: https://CRAN.R-project.org/package=DescTools

22. Zeileis A, Hothorn T. Diagnostic Checking in Regression Relationships. R News. 2002;2: 7–10.

23. R Core Team. R: A Language and Environment for Statistical Computing. Vienna, Austria: R Foundation for Statistical Computing; 2023. Available: https://www.R-project.org/

**ENGLISH ANSWER**

We sincerely appreciate your insightful comments aimed at improving our analysis. In response, we have incorporated the calculation of the Akaike Information Criterion (AIC) for each adjusted model and enhanced our model-building strategy by including likelihood ratio tests. These updates are detailed in the “Methods” and “Results” sections, respectively, as outlined below:

In “Methods” section

Line 297: “Then, remaining variables were entered into a model without interactions following a direct approach. Subsequently, predictors with the weakest evidence of association were iteratively removed, assessing changes in estimated coefficients for remaining variables. This process was complemented by likelihood ratio tests, performed using the “lrtest” function from the “lmtest” package in R [22]. If estimated coefficients varied by 5% or more and the likelihood ratio test was significant (p-value < 0.05), the predictor was deemed contributory to the final model and reintroduced. Otherwise, it was considered non-contributory and discarded. This process continued until only predictors with strong evidence of association and significant likelihood ratio test remained. Subsequently, interaction effects between each drug and COVID-19 booster were introduced into the model. Interaction lacking evidence of association were sequentially removed based on the estimated P values and supported by non-significant likelihood ratio tests. The elimination of interaction effects stopped once only those with strong evidence of association remained in the final adjusted model. Furthermore, the Akaike Information Criterion (AIC) was calculated for each adjusted model assessed using the “AIC" function from the “stats” package in R [23].”

In “Results” section

Line 420: “When evaluating the complete model without interaction effects, it was found that all predictors presented strong evidence of association, and these provided significant information to the model through likelihood ratio test (Appendix S2). Also, comorbidities minimally impacted the estimated coefficients (never exceeding 5%) but were retained as potential confounders. Therefore, it was decided not to remove any variable from the model and to continue evaluating the interaction effects between each medication consumption (vitamin C and acetylsalicylic acid) and COVID-19 booster. As a result, it was found that no interaction effect presented strong evidence of association and did not provide significant information to the model through likelihood ratio test (Appendix S2). Thus, it was decided to maintain the model without interaction effects as the final adjusted model which presented an AIC of 164.6.”

The model-building process—including the results of the likelihood ratio tests and the estimated AIC values—is now presented in a new supplementary file titled *Appendix S2*, which has been included with this submission.

In addition, regarding the assessment of potential collinearity, we initially estimated the Phi coefficient between the variables considered in the adjusted model. However, as the variable “self-perceived risk of COVID-19” (which comprises four categories) was included in the current analysis, we have updated our collinearity assessment using Cramer’s V coefficient. This measure follows a similar rationale to the Phi coefficient but is more appropriate for variables with more than two categories. To improve transparency, we have also revised the “Methods” and “Results” sections to clarify the explanation of the collinearity assessment, as outlined below:

In “Methods” section

Line 292: “Regarding the adjusted model building, potential collinearity among the initial variables considered was evaluated using a correlation matrix based on Cramer's V coefficient, calculated with the “CramerV” function from the “DescTools” package in R [21]. If a group of variables presented at least a weak relationship (Cramer’s V ≥ 0.2), the variable with weaker evidence of association with the SARS-CoV-2 diagnosis was discarded.”

In “Results” section

Line 408: “N-acetylcysteine consumption was also excluded based on the detected collinearity with vitamin C through correlation matrix analysis (S3 Table). The remaining variables did not exhibit potential collinearity, as all Cramér’s V coefficients were below 0.2. Thus, the initial model…”

In order to address this suggestion, the following additional references have been incorporated into the revised version of the manuscript:

21. Signorell A. DescTools: Tools for Descriptive Statistics. R Project; 2023. Available: https://CRAN.R-project.org/package=DescTools

22. Zeileis A, Hothorn T. Diagnostic Checking in Regression Relationships. R News. 2002;2: 7–10.

23. R Core Team. R: A Language and Environment for Statistical Computing. Vienna, Austria: R Foundation for Statistical Computing; 2023. Available: https://www.R-project.org/

Best regards,

Javier Guitian
