# Supplementary material for: Association between self-administrated prophylactics and SARS-CoV-2 infection among traditional market vendors from the Central Highlands of Peru: A nested case-control study
Source: PLoS One. 2025 Jul 11;20(7):e0327746. doi: 10.1371/journal.pone.0327746 (PMC12250348; doi:10.1371/journal.pone.0327746)
Supplement: Attachment — Submitted filename: Response_to_reviewers_auresp_2.docx [file pone.0327746.s008.docx]

Dear Editor,

We sincerely thank the reviewers and the editorial team for their thoughtful comments and suggestions, which have substantially contributed to improving our manuscript (PONE-D-25-09334R1). In response, we have carefully revised the manuscript and are pleased to submit the updated version for your consideration.

All comments have been addressed in the accompanying document. Our responses are presented in green, and the corresponding edits to the manuscript are shown in red.

**REVIEWERS’ COMMENTS**

REVIEWER #1

The manuscript addresses the previously raised comments; however, I have a few minor suggestions for further improvement:

1. Recruitment and Selection Bias: The manuscript should clearly explain how participants were recruited into the program. This aspect, while noted in the limitations, may represent a potential source of selection bias, and a more detailed description would help the reader better understand the possible direction and magnitude of this bias.

Was a random sampling method employed, or were all eligible individuals included? If not all were included, please report the rejection rate and provide any relevant details on the selection process.

We appreciate the suggestion to further clarify the recruitment process employed in our study. In response, we have included additional details in the Methods section to provide a more comprehensive description of how participants were recruited. The revised text reads as follows:

Line 110: “The program comprised of three sequential phases conducted before, during, and after the third wave of SARS-CoV-2 infections. During the first phase, from August to December 2021, the program worked with market vendors to establish its objectives, and consultation rooms were set up in locations within each market as designated by the vendors. Also, health workers assigned to each market oversaw participant registration and monitoring throughout the program. Recruitment was conducted through direct communication from market authorities to all workers in each market. Participation was voluntary and based on convenience sampling, as individuals self-enrolled by presenting themselves at the consultation room from their respective markets. No formal rejections were recorded, although not all eligible workers chose to participate. Finally, registration involved an interviewer-administered questionnaire, a clinical examination, and a nasopharyngeal swab for an antigen SARS-CoV-2 test (Roche Diagnostics SL, Barcelona, Spain), along with voluntary validation of COVID-19 vaccination status using individual vaccination cards given by the Peruvian Ministry of Health.”

Finally, we have also addressed the potential selection bias associated with the recruitment approach used in this study by revising and reorganizing the first paragraph of the limitations as follows:

Line 549: “Our study has limitations. First, the results found in our study may not be fully generalizable, as participants were exclusively traditional market vendors, and recruitment was conducted through a non-random, convenience-based approach. Specifically, participation was voluntary and limited to individuals who actively approached the registration office in response to announcements made by market authorities. This self-selection process may have led to selection bias, as those more interested or concerned about COVID-19 might have been more likely to enroll. In addition, no formal records of refusals during the first phase of the health program, further limiting the ability to assess the direction or magnitude of this potential bias. Nonetheless, according to the last National Census of Food Markets in 2016 [48], Market A reported approximately 600 operational stalls and Market B reported 1,000. In comparison, our study initially recruited 224 vendors from Market A and 215 from Market B, suggesting that a substantial fraction of the total vendor population was recruited although the obtained sample could be potentially biased. Further, traditional markets could still serve as potential SARS-CoV-2 sentinel surveillance sites, given their constant interaction with the general population.”

In order to address this suggestion adequately, we have added the following reference in the final version of this manuscript:

48. Instituto Nacional de Estadística e Informática. Censo Nacional de Mercados de Abasto. 2016: Resultados a nivel nacional [National Census of Food Markets 2016: National-level results]. Lima, Peru: Instituto Nacional de Estadística e Informática (INEI); 2017. Available: https://www.inei.gob.pe/media/MenuRecursivo/publicaciones_digitales/Est/Lib1448/libro.pdf

2. Questionnaire Validation: It is essential to specify whether the questionnaire used in the study has been previously validated. If so, please provide details on the validation process, including the population and setting in which it was validated. If it is an ad hoc instrument, a discussion on its development and limitations is necessary.

Thank you for highlighting the need for greater clarity regarding the validation process of the questionnaire used in our study. We have now specified that it was an ad hoc instrument, developed specifically for the purposes of this research. The validation process involved a content evaluation conducted by a panel of subject-matter experts and a pilot study conducted in an external market, as follows:

Line 207: “A structured interviewer-administered questionnaire was employed to collect data on participant’s characteristics. The questionnaire was developed by the authors after a detailed review of the literature. As it was an ad hoc instrument, its content validity was evaluated by a panel of subject-matter experts appointed during the ethical review process. The experts assessed the questionnaire for clarity, relevance, and alignment with the study objectives. Minor revisions were made based on their feedback. Finally, a pilot test was conducted with vendors from an external traditional market to evaluate participants’ comprehension and ensure the questions were appropriately understood. Additional minor adjustments were made based on insights gained from the pilot study to enhance clarity and consistency.”

Also, we have discussed on its development and limitations as follows:

Line 595: “Sixth, the questionnaire used was an ad hoc instrument developed specifically for the purposes of this study. Although it underwent expert review as part of the ethical assessment process, and was piloted in a group of vendors from an external traditional market to evaluate clarity and participant understanding, the questionnaire may still present limitations in its psychometric robustness and could introduce measurement bias in self-reported behaviours.”

REVIEWER #2

The statistical procedure was duly considered and clarified. The results were explained, and collinearity was assessed using Cramér’s V test.

We sincerely appreciate the valuable suggestions provided during the previous revision round, which have significantly contributed to enhancing the quality of this manuscript.

Best regards,

Javier Guitian
